# SIVcpz closely related to the ancestral HIV-1 is less or non-pathogenic to humans in a hu-BLT mouse model

**DOI:** 10.1038/s41426-018-0062-9

**Published:** 2018-04-04

**Authors:** Zhe Yuan, Guobin Kang, Lance Daharsh, Wenjin Fan, Qingsheng Li

**Affiliations:** 1 School of Biological Sciences, Nebraska Center for Virology, University of Nebraska-Lincoln, Lincoln, NE 68583 USA; 2 Present Address: National Institute of Allergy and Infectious Diseases, National Institute of Health, Bethesda, MD 20892 USA

## Abstract

The HIV-1 pandemic is a consequence of the cross-species transmission of simian immunodeficiency virus in wild chimpanzees (SIVcpz) to humans. Our previous study demonstrated SIVcpz strains that are closely related to the ancestral viruses of HIV-1 groups M (SIVcpzMB897) and N (SIVcpzEK505) and two SIVcpz lineages that are not associated with any known HIV-1 infections in humans (SIVcpzMT145 and SIVcpzBF1167), all can readily infect and robustly replicate in the humanized-BLT mouse model of humans. However, the comparative pathogenicity of different SIVcpz strains remains unknown. Herein, we compared the pathogenicity of the above four SIVcpz strains with HIV-1 using humanized-BLT mice. Unexpectedly, we found that all four SIVcpz strains were significantly less pathogenic or non-pathogenic compared to HIV-1, manifesting lower degrees of CD4+ T-cell depletion and immune activation. Transcriptome analyses of CD4+ T cells from hu-BLT mice infected with SIVcpz versus HIV-1 revealed enhanced expression of genes related to cell survival and reduced inflammation/immune activation in SIVcpz-infected mice. Together, our study results demonstrate for the first time that SIVcpz is significantly less or non-pathogenic to human immune cells compared to HIV-1. Our findings lay the groundwork for a possible new understanding of the evolutionary origins of HIV-1, where the initial SIVcpz cross-species transmission virus may be initially less pathogenic to humans.

## Introduction

The HIV-1 pandemic is a consequence of cross-species transmission of simian immunodeficiency virus from wild chimpanzees (SIVcpz) to humans^1–3^. At least four independent cross-species transmissions of SIVs from chimpanzees and gorillas in Africa to humans have occurred, which led to infections from HIV-1 groups M, N, O, and P in humans^1–4^. Although the HIV-1 pandemic began in the early 1980s, the SIVcpz spillover from chimpanzees into humans began much earlier. It was estimated that the date of the most recent common ancestor (MRCA) of HIV-1 group M was around 1908 (1884−1924)^5^, whereas the date of the MRCA of the shared HIV-1 group M and SIVcpz was estimated as 1853 (1799−1904)^6^ or 1876 (1847−1907)^7^. Thus, the most likely time period of cross-species transmission of SIVcpz as the ancestral HIV-1 group M virus to humans is between 1853 and 1908. Consistent with these estimates, the earliest spread of HIV-1 within humans was reported around 1920 in Kinshasa^8^. However, there are no recorded AIDS-related deaths before the first documented HIV-1 infection in the Congo in 1959, whose actual cause of death remains unknown^9^. These data suggest that SIVcpz early cross-species infections of humans appear to be clinically “silent” for at least five decades. Many questions regarding the evolutionary history of HIV-1 and the pathogenicity of SIVcpz to humans remain unanswered. These questions are fundamentally important for understanding the evolutionary origins of the devastating pandemic of HIV-1 infections and for predicting the likelihood of the occurrence of another HIV-1-like infection in humans, as more than 30 African non-human primate (NHP) species are still infected with more than 40 different strains of SIVs^3^. Furthermore, there has been an increase in human exposure to NHPs^10^, and there is recent evidence of continuing cross-species transmissions of SIV from monkeys^10,11^ and great apes^4,12^ to humans.

Humanized-BLT mice infected with HIV-1 can recapitulate the pathogenesis of HIV-1 infection of humans. It has been extensively documented that hu-BLT mice infected with different strains of HIV-1, including JRCSF^13–15^, MNp^13^, NL4-3^13^, ADA^16^, and transmitted/founder HIV-1^17^, all result in CD4+ T-cell depletion, a hallmark of HIV pathogenicity and the foundation for using hu-BLT mice as a model of HIV infection of humans. Our previous study demonstrated SIVcpz strains that are closely related to the ancestral viruses of HIV-1 groups M (SIVcpzMB897) and N (SIVcpzEK505) and two lineages of SIVcpz that are not associated with any known HIV-1 infection in humans (SIVcpzMT145 and SIVcpzBF1167), and all can readily infect and robustly replicate in humanized-BLT mice^18^. In the current study, we compared the pathogenicity of these four SIVcpz viruses with pandemic HIV-1 using the hu-BLT mouse model. Using reasoning based on our previous finding that SIVcpz replicated well in hu-BLT mice, we initially hypothesized that SIVcpz would cause similar levels of CD4 T-cell depletion and immune activation as HIV-1. However, our results contradicted our initial hypothesis. We found, unexpectedly, that the SIVcpz strains that are closely related to the ancestral viruses of groups M and N, as well as the lineages of SIVcpz that are not associated with any known HIV-1 infections in humans, are all significantly less pathogenic or non-pathogenic in hu-BLT mice compared with HIV-1, manifesting significantly lower degrees of CD4+ T-cell depletion and cell activation compared with uninfected controls and HIV-1-infected animals. RNA-Seq analyses also revealed that CD4+ T cells from SIVcpz-infected animals had lower expression levels of genes related to cell death, cell cycle arrest, cytokine/cytokine receptor interactions, inflammatory responses, and cell activation, as well as higher expression levels of genes related to cell survival and promotion of the cell cycle compared to CD4+ T cells from HIV-1-infected animals.

This study, for the first time, has experimentally recapitulated the pathogenicity of SIVcpz infection of human cells in vivo and has demonstrated that SIVcpz strains are less pathogenic or non-pathogenic in humans compared with HIV-1 using a hu-BLT mouse model. Our conceptual framework and experimental system are valuable for gauging the potential risk of SIVs and other zoonotic pathogens spilling over to humans to cause another HIV-1-like or other infectious disease. Our data support the possibility of a new model for the evolutionary origins of HIV-1, where the SIVcpz cross-species transmission virus may be initially less pathogenic and gains virulence over time within the human population.

## Materials and methods

### Virus stock preparation

Virus stocks were generated as previously reported^18^. Briefly, 60 µg of plasmid DNA from infectious molecular clones of SIVcpz (SIVcpzMB897, SIVcpzEK505, SIVcpzMT145, and SIVcpzBF1167) and HIV-1_SUMA_ were transfected into 293T cells. After 48 h of transfection, culture supernatant was collected from each flask and filtered through a 0.45-micron filter. Thirty-five milliliters of filtered medium was loaded into each Ultra-Clear™ Tube (Beckman Coulter) for ultracentrifugation. Virus ultracentrifugation was conducted with an Optima L-100X ultracentrifuge and an SW 32 Ti rotor (Beckman Coulter) at 25,000 rpm for 90 min at 4 °C. The supernatant was discarded, and the pellet was resuspended into 1 ml of fresh medium, aliquoted into 200 µl in sterile screw-cap vials and stored at −150 °C. Virus stocks were titrated on the TZM-bl reporter cell line with the X-Gal Staining Kit (Genlantis). Titers are expressed as TZM-bl infectious units (IU) per ml.

### Generation of hu-BLT mice

Hu-BLT mice were generated as reported at the University of Nebraska-Lincoln according to Institutional Animal Care and Research Committee-approved protocols^17^. Briefly, 6- to 8-week-old female NSG mice (NOD.Cg-Prkdcscid Il2rgtm1Wjl/SzJ, Cat# 005557, the Jackson Laboratory) were irradiated at a dose of 12 cGy/g body weight with an RS200 X-ray irradiator (RAD Source Technologies, Inc., GA) and were implanted with one piece of thymic tissue sandwiched between two pieces of human fetal liver tissue under the murine left renal capsule. Within 6 h of surgery, the mice were injected via the tail vein with 1.5–5×10^5^ CD34^+^ hematopoietic stem cells isolated from human fetal liver tissues. Human fetal liver and thymus tissues were procured from Advanced Bioscience Resources (Alameda, CA). After 9−12 weeks, human immune cell reconstitution in the peripheral blood was measured by a FACS Aria II flow cytometer (BD Biosciences, San Jose, CA) using antibodies against mCD45-APC, hCD45-FITC, hCD3-PE, hCD19-PE/Cy5, hCD4-Alexa 700, and hCD8-APC-Cy7 (Cat#103111, 304006, 300408, 302209, 300526, and 301016, respectively, BioLegend, San Diego, CA). Raw data were analyzed with FlowJo (version 10.0, FlowJo LLC, Ashland, OR). All mice used in this study had high human immune reconstitution with a ratio of hCD45^+^ cells to a combination of hC45^+^ cells and mCD45^+^ cells in peripheral blood greater than 50%. The mice were randomly assigned into experimental groups with similar immune reconstitution levels (Table 1).

**Table 1:** Hu-BLT mice used in this study

Animal ID	% hCD45^+^/(hCD45^+^ and mCD45^+^ cells)	%hCD3^+^ in hCD45^+^	%hCD8^+^ in hCD45^+^hCD3^+^ cells	%hCD4^+^ in hCD45^+^ hCD3^+^ cells	Experimental group
HM658	50.3	54.5	19.1	77.2	HIV-1
HM660	50.1	80.9	16.4	80.2	
HM662	63.9	40.4	21.5	74	
HM670	69.3	75.1	17.2	79.4	
HM695	57	55.9	13.5	85.2	
Avg	58.12	61.36	17.54	79.2	
HuM 623	79.9	64.6	17.8	81.6	MB897
HuM 624	84.1	63.9	14.5	84.7	
HuM 627	88.1	67	13.3	85.7	
HuM 628	62.7	57.8	15.6	83.1	
HuM 629	75	67.5	12.1	86.7	
Avg	77.96	64.16	14.66	84.36	
HuM 632	59.2	62.3	12.7	82.8	EK505
HuM 634	76.3	70.4	11.4	87.6	
HuM 635	50.7	51.2	20.9	77.3	
HuM 639	60	75	12.7	86.1	
HuM 640	79.7	73.6	13.5	85.7	
Avg	65.18	66.5	14.24	83.9	
HM709	55.1	64.5	12.7	80.9	MT145
HM714	80.4	92.7	16.9	80.2	
HM718	50.1	88	15.3	80.3	
HM719	55.9	79.6	13	83.3	
HM723	50.1	85.9	15.1	82.5	
Avg	58.32	82.14	14.6	81.44	
HM698	56.4	65	12.1	82.2	BF1167
HM702	50.6	56.5	16.5	75.6	
HM703	76.8	77.8	13.6	83.3	
HM706	68.7	64.9	13.7	82	
HM708	50.8	68.2	15.1	79.8	
Avg	60.66	66.48	14.2	80.58	
HuM 620	79.5	58.2	20.3	78.5	Negative
HuM 621	82.1	57	20.4	78.9	
HuM 622	86.4	69.4	11.7	87.3	
Avg	82.67	61.53	17.47	81.57	

### SIVcpz and HIV-1 infections of hu-BLT mice

Female hu-BLT mice with high immune reconstitution were randomly divided into six groups (Table 1). The mice in each infection group (*n* = 5/each group) were inoculated intraperitoneally (IP) with 3 ± 0.2×10^4^ IU of SIVcpzMB897, SIVcpzEK505, SIVcpzMT145, SIVcpzBF1167, or HIV-1_SUMA_^19^. Mice without inoculation were used as uninfected controls. Peripheral blood was collected bi-weekly post-inoculation. At 16 weeks post-inoculation (wpi), mice were euthanized, and the spleens were collected. Half of the spleen tissue was fixed in SafeFix II (Fisher Scientific) for immunohistochemical staining (IHCS), and the other half of the spleen tissue was used for single cell isolation. The lymphocytes were separated through Ficoll first. The human lymphocytes were further sorted using the EasySep™ Mouse/Human Chimera Isolation Kit (Catalog #19849, STEMCELL Technologies) and an EasySep™ Magnet (Catalog #18000, STEMCELL Technologies).

### Plasma viral load

Plasma viral loads (pVL) were measured as previously reported^18^. Briefly, viral RNA (vRNA) was extracted using a QIAamp Viral RNA Mini kit (Qiagen). The pVL were determined using qRT-PCR on a C1000 Thermal Cycler and the CFX96 Real-Time system (Bio-Rad) with the TaqMan Fast Virus 1-Step Master Mix (Life Technologies).

### Flow cytometry

Peripheral blood and human lymphocytes from the spleen were measured by a FACS Aria II flow cytometer (BD Biosciences, San Jose, CA). Cells were blocked using Human TruStain FcX and Mouse TruStain fcX Antibodies (Cat# 422302 and 101320, respectively, BioLegend) prior to staining. Then, cells were stained using antibodies against hCD45-APC/Cy7, hCD3-FITC, hCD4-PE/Cy7, hHLA-DR-APC, and hCD38-PE (Cat#304014, 300406, 344612, 307610, and 356604, respectively, BioLegend) as well as hCD8a-PE/Cy5.5 (Cat# 9536-16, Southern Biotech). Data were analyzed with FlowJo (version 10.0, FlowJo LLC, Ashland, OR).

### Immunohistochemical staining (IHCS), immunofluorescent staining (IF), and quantitative image analysis (QIA)

Both IHCS and IF were conducted primarily by following our previously published methods^20^ with the following modifications. For IHCS, rabbit anti-hCD4 monoclonal antibody (EPR6855, 1:100 dilution, Abcam) was used as the primary antibody, and anti-rabbit/HRP polymers from the MultiVision Polymer Detection System kit (Thermo Scientific, Cat# TL-012-MARH) were used to visualize CD4 signals as blue. The sections were counterstained with eosin. For IF, rabbit anti-hCD4 monoclonal antibody (EPR6855, 1:200 dilution, Abcam) and sheep anti-hKi67 polyclonal antibody (AF7617-SP, 1:150, Novus Biologicals) were used as the primary antibodies. Alexa Fluor^®^ 647-conjugated donkey anti-sheep IgG and Alexa Fluor^®^ 488-conjugated donkey anti-rabbit IgG were used as the secondary antibodies. DAPI was used for counterstaining. To reduce the autofluorescence in spleen tissues, tissue sections were subjected to Sudan Black treatment with 0.3% Sudan Black B (Catalog# 190160250, ACROS Organics) in 70% ethanol stirred in the dark for 2 h before mounting. For both IHCS and IF, isotype control antibody or/and without primary antibody was used as the negative control. For QIA of CD4+ T cells in IHCS sections, CD4+ T cells were quantified using a positive pixel count algorithm in Aperio’s Spectrum Plus analysis program (version 9.1; Aperio ePathology Solutions). QIA of the percentage of Ki67+ cells within CD4+ T cells in IF sections was performed manually using the Scanscope marker tool after confocal images were uploaded into Aperio’s Spectrum Plus analysis program (version 9.1; Aperio ePathology Solutions).

### RNA extraction and mRNA-Seq

RNA-Seq was conducted on human CD4+ T cells derived from spleen tissues of SIVcpzMB897-, SIVcpzBF1167-, and HIV-1-infected animals (*n* = 3/each group). Human CD4+ T cells were negatively sorted by magnetic beads using a human CD4+ T Cell Isolation Kit (Catalog #130-096-533, Miltenyi Biotec). Total RNA was extracted from sorted human CD4+ T cells using an RNeasy Plus Mini Kit (Qiagen). A SMARTer^®^ Stranded Total RNA-Seq Kit (Pico Input Mammalian) was used for library preparation (Clontech Laboratories, Inc). Libraries were used for sequencing on an Illumina HiSeq 2500 Rapid Mode at the University of Minnesota Genomics Center (Minneapolis, MN). Each of the nine sequenced samples generated more than 240 million 100-bp paired-end pass filter (PF) reads. The average quality scores were above Q30 for all the PF reads. All expected barcodes were detected and balanced.

### Transcriptome analysis

Paired-end fastq files were submitted to FastQC^21^ analysis for quality control. Low quality reads were then trimmed or removed by Trimmomatic^22^ to produce a set of paired and unpaired fastq files. The resulting paired and unpaired reads for both sets of paired-end sequencing files were mapped to the reference genome using Tophat 2.1 and the Illumina iGenome Bowtie index Ensembl GRch37. Cufflinks 2.2 was used to estimate the relative abundance of the transcripts based on the previously mapped genome reads^23^. Cuffmerge was used to combine the cufflinks output. Cuffdiff was used to determine significant differences in transcript expression between SIVcpz and SUMA. The gene expression difference list was filtered by adjusted *q* value < 0.0678 and log_2_ fold change >2. The gene function analysis and category annotation were based on QIAGEN’s Ingenuity Pathway Analysis (IPA, QIAGEN). Heatmaps were created using the gplots R package and heatmap.2 function^24^. The Cuffdiff log2 fold change output from the comparison of SIVcpz (BF & MB) with SUMA was plotted with a log_2_ range from less than −5 to greater than 5.

### Statistics

Two-way ANOVA with Bonferroni post-tests were used to test for significant differences in pVL, CD4+ T-cell depletion, CD4+ T cells, and CD4+ T-cell activation for SIVcpz- and HIV-1_SUMA_-infected animals at different time points post-inoculation. All tests were performed using GraphPad Prism software (GraphPad software, San Diego, CA, USA). *P* < 0.05 was considered significant.

### Data availability

The raw sequencing data can be found at the NCBI Sequence Read Archive (SRA) under accession number SRX7562306.

## Results

### Viral replication kinetics of SIVcpz and HIV-1 in hu-BLT mice

**Fig. 1:**
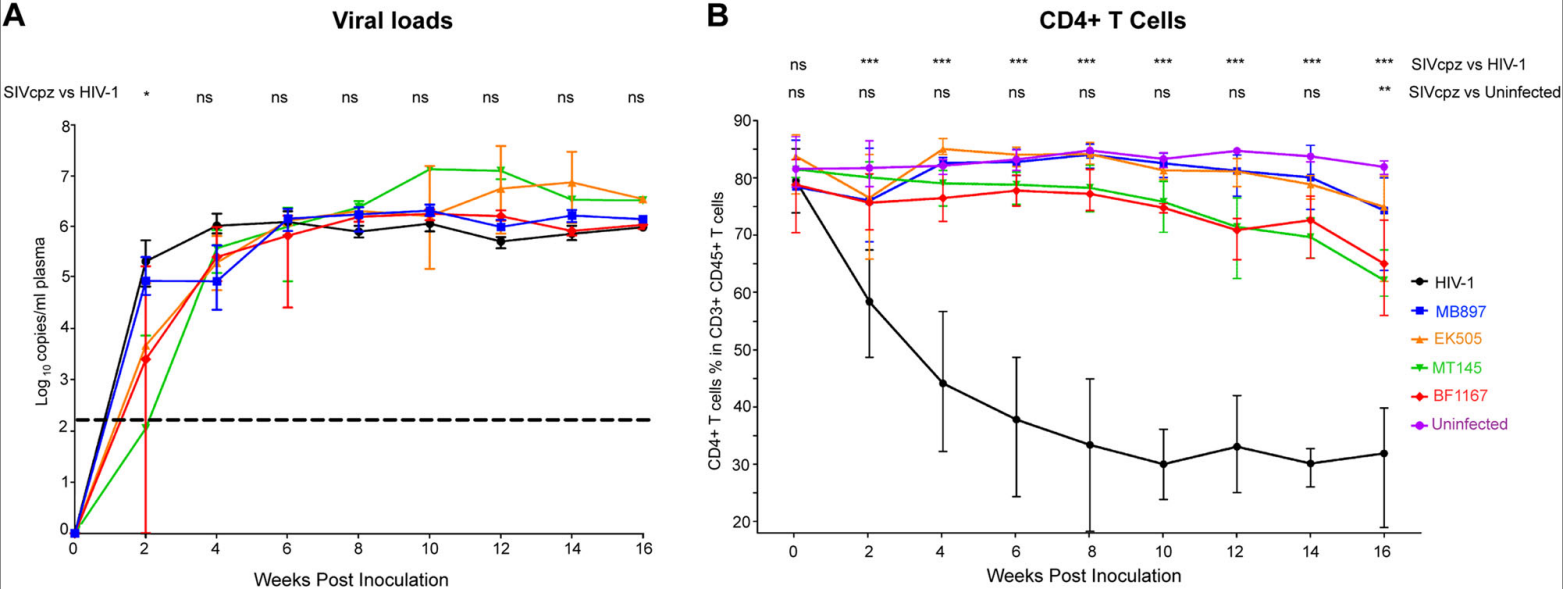
Plasma viral load and CD4+ T-cell kinetics in hu-BLT mice infected with HIV-1 or one of four different strains of SIVcpz. (**A**) Mean plasma VL kinetics over the course of 16 weeks pi. Five groups of hu-BLT mice (*n* = 5/each group) were inoculated with a high dose of SIVcpz closely related to the ancestral viruses of HIV-1 groups M (SIVcpzMB897) and N (SIVcpzEK505), two lineages of SIVcpz strains that have not been associated with any known HIV-1 infections in humans (SIVcpzMT145 and SIVcpzBF1167), and HIV-1. Each group is color coded. The dashed line indicates the detection limit of pVL. Statistical significance is indicated with stars. NS non-significance, *<0.05, **<0.01, ***<0.001. (**B**) CD4+ T-cell depletion in four different SIVcpz- and HIV-1-infected hu-BLT mice. The CD4+ T-cell percentage in the total T cells of five groups of infected hu-BLT mice were quantified. Each group is color coded. Statistical significance is indicated with stars. NS non-significance, *<0.05, **<0.01, ***<0.001

There are two subspecies of chimpanzees, *Pan troglodytes troglodytes* (Ptt) and *Pan troglodytes* s*chweinfurthii* (Pts), in Africa that are the natural hosts for SIVcpz. Ptt chimpanzees are distributed throughout southern Cameroon, Gabon, and the Republic of Congo and are the natural host for the ancestral viruses of HIV-1 groups M and N^1–3^. Pts chimpanzees are distributed throughout the Democratic Republic of the Congo and countries to the East, and SIVcpz virus from this host has not been found in human infection^1–3^. Our previous work showed robust replication of all four SIVcpz strains in hu-BLT mice with similar viral kinetics compared to HIV-1^18^. In this study, we compared the plasma viral load kinetics of four SIVcpz strains, including SIVcpz strains that are closely related to the ancestral viruses of HIV-1 groups M (SIVcpzMB897) and N (SIVcpzEK505) and two lineages of SIVcpz that are not associated with any known HIV-1 infections in humans (SIVcpzMT145 and SIVcpzBF1167), and current pandemic HIV-1 using hu-BLT mice. As shown in Fig. 1a, consistent with our previous results, all four SIVcpz strains replicated at high levels from the acute to the chronic stage of infection and had similar pVL kinetics to HIV-1, regardless of whether they were derived from Ptt (SIVcpzMT145) or Pts (SIVcpzBF1167) chimpanzees. There were no significant differences between pVL during SIVcpz and HIV-1 infection for all the measured time points, except for 2 weeks post-infection (wpi). While SIVcpzMB897 was not significantly different compared to HIV-1 at 2 wpi, the other three SIVcpz strains were significantly different from HIV-1 (*P* < 0.05) (Fig. 1A).

### CD4+ T-cell depletion in peripheral blood during SIVcpz and HIV-1 infection

Next, we investigated the pathological consequences of the SIVcpz infection of humans using the hu-BLT mouse model. We quantified CD4+ T cells as a parameter of pathogenicity in SIVcpz- and HIV-1-infected hu-BLT mice using flow cytometry. As shown in Fig. 1B, HIV-1-infected BLT mice had a significant depletion of CD4+ T cells, which is consistent with previously published work where hu-BLT mice had been infected with different strains of HIV-1^13−15,17^. CD4+ T cells were significantly depleted by the first sampled time point of 2 wpi, reaching a nadir at 10 wpi and remaining at this low level until the end of the study at 16 wpi. In contrast, all four strains of SIVcpz surprisingly did not cause a significant decline of CD4+ T cells until 16 wpi in infected hu-BLT mice (15–20% less than 0 wpi), which contradicts our initial hypothesis that SIVcpz would cause a similar level of CD4 T-cell depletion as HIV-1. Compared to the HIV-1-infected group, the CD4+ T-cell counts in the SIVcpz-infected group were significantly higher (*p* < 0.001) at all time points. Compared to the uninfected control group, there were no significant differences in the CD4+ T-cell counts in the SIVcpz-infected group until the final time point of the study at 16 wpi (*p* < 0.01). There were no significant differences between any of the SIVcpz strains in terms of the CD4+ T-cell depletion. Thus, we found that SIVcpz infection of hu-BLT mice does not result in a significant decrease of CD4+ T cells despite high viremia.

### CD4+ T-cell depletion in the secondary lymphatic tissues of SIVcpz and HIV-1 infection

**Fig. 2:**
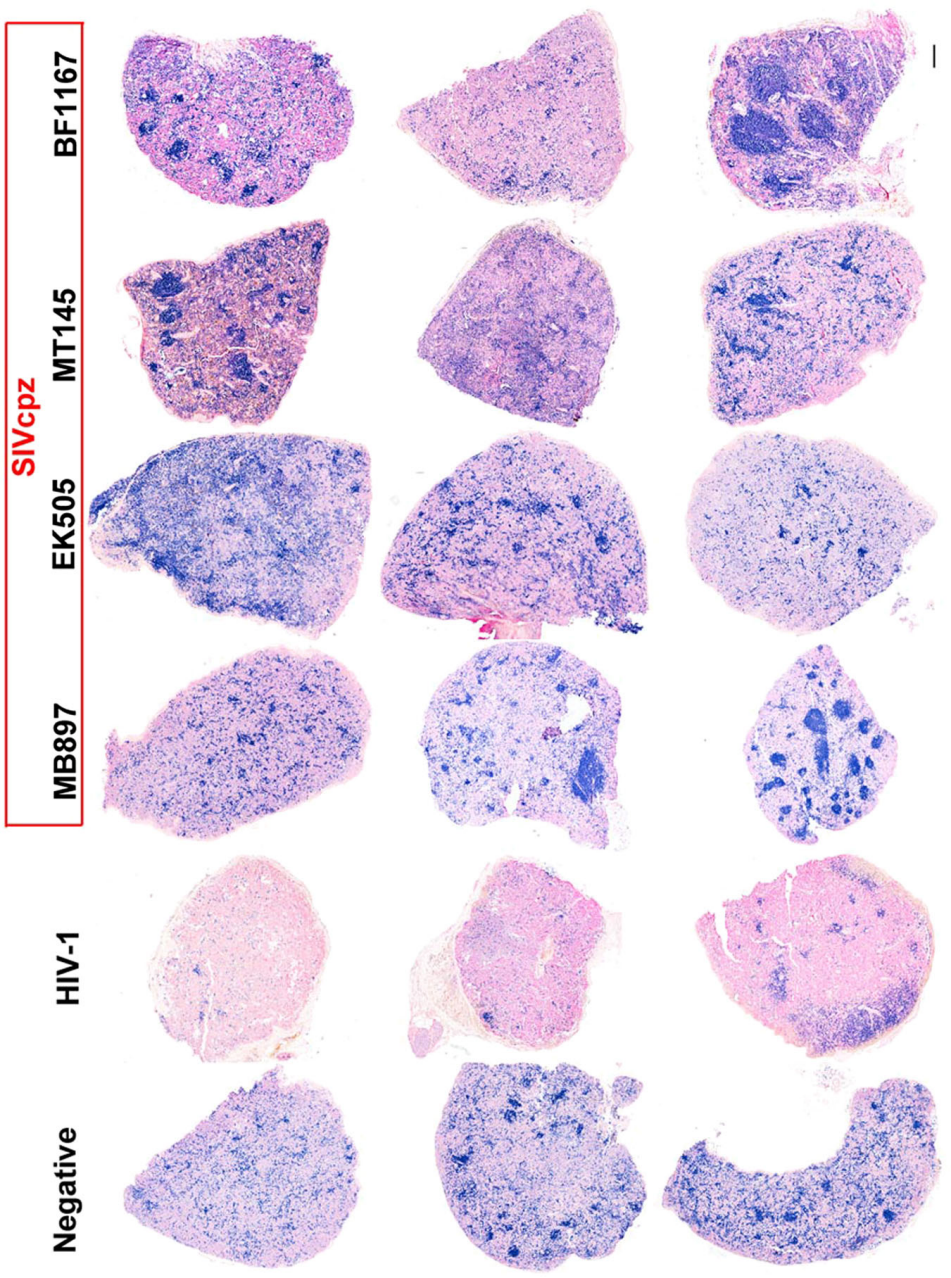
CD4+ T cells in splenic tissues of SIVcpz- and HIV-1-infected hu-BLT mice at 16 wpi. CD4+ T cells in whole sections of splenic tissues were detected using immunohistochemical staining. CD4+ T cells are stained blue. Scale bar, 200 µm

**Fig. 3:**
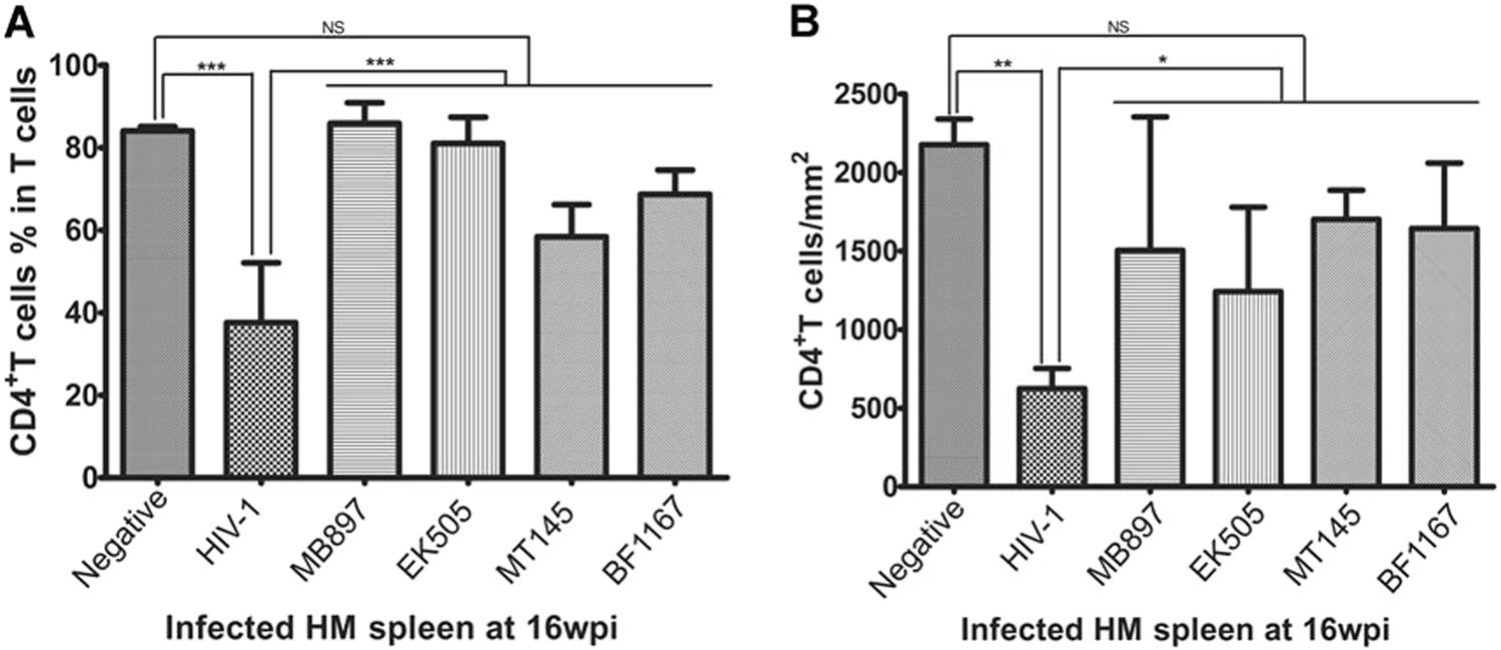
Quantification of CD4+ T cells in splenic tissues of SIVcpz- and HIV-1-infected hu-BLT mice. (**A**) The CD4+ T-cell percentage in the total T cells from splenic tissues of each group (*n* = 5) were quantified using flow cytometry at 16 wpi. **(B)** CD4+ T-cell counts per square millimeter of spleen tissues were quantified after IHCS at 16 wpi. Statistical significance is indicated with stars. NS non-significance, *<0.05, **<0.01, ***<0.001

To further compare CD4+ T cells in lymphoid tissues of SIVcpz- versus HIV-1-infected hu-BLT mice, we quantified CD4+ T cells in the secondary lymphatic tissues, where the majority of the CD4+ T cells reside, using flow cytometry as well as IHCS and QIA. At 16 wpi, the animals were killed, a portion of the spleen tissue was fixed for IHCS and QIA, and another portion was used for single cell isolation followed by flow cytometry. Figure 2 shows human CD4+ T cells in the splenic tissues of the five different groups. There was a clear depletion of CD4+ T cells in the spleen tissues of HIV-1-infected mice compared to SIVcpz-infected and uninfected animals, both of which contained significantly more CD4+ T cells. Both methods of CD4+ T-cell quantification in spleen tissues, including flow cytometry (Fig. 3a) as well as IHCS and QIA (Fig. 3b), consistently demonstrated much less CD4+ T-cell depletion during SIVcpz infection compared with HIV-1 infection. While there was a significant difference between HIV-1-infected animals and SIVcpz-infected or uninfected animals, SIVcpz-infected and uninfected animals did not differ significantly (*p* values as shown in Fig. 3).

### Immune activation in SIVcpz and HIV-1 infection

**Fig. 4:**
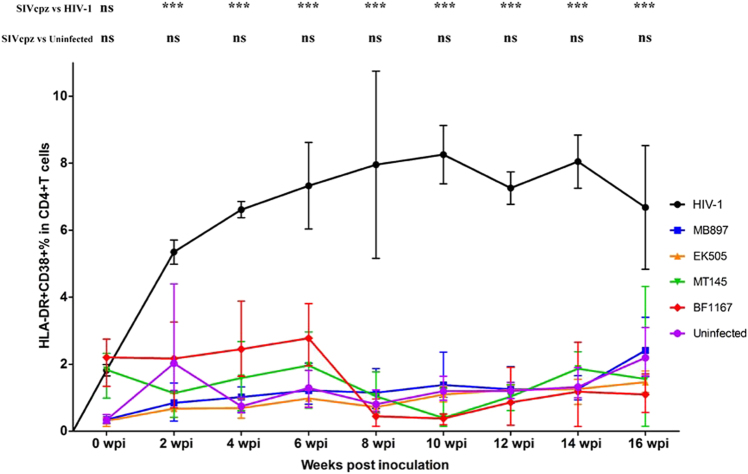
CD4+ T-cell activation in SIVcpz- and HIV-1-infected hu-BLT mice. CD38+ HLA-DR+ ratios in the CD4+ T cells of the five groups (*n* = 5/each group) were measured. Each group is color coded. Statistical significance is indicated with stars. NS non-significance, *<0.05, **<0.01, ***<0.001

**Fig. 5:**
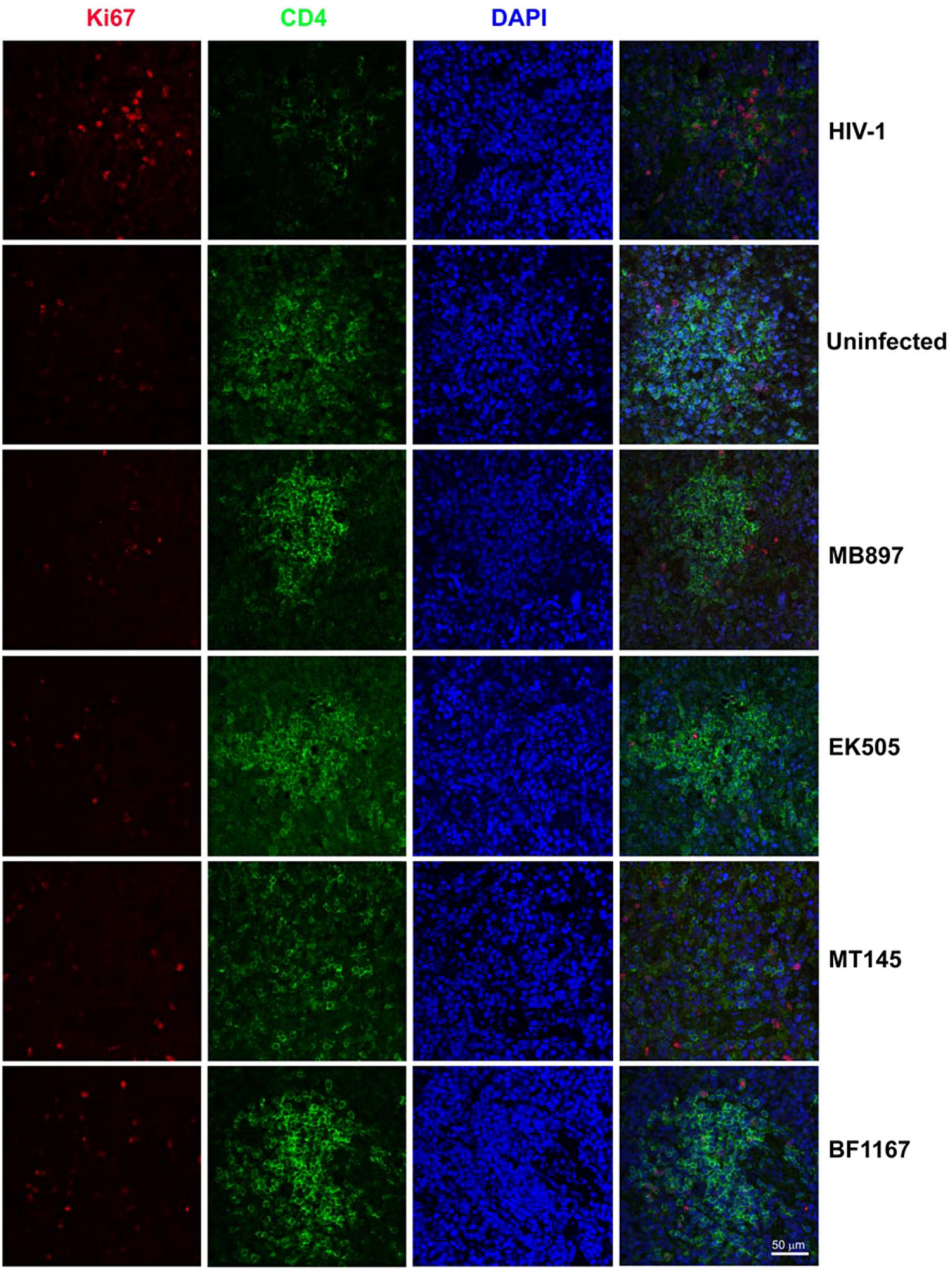
Immune activation in splenic tissues of SIVcpz- and HIV-1-infected hu-BLT mice detected using immunofluorescent staining of human CD4 and Ki67. Human CD4+ T cells are shown in green, human Ki67+ cells in red, and DAPI in blue. Each row is labeled by the virus strain used for infecting the animals. Scale bar, 50 µm

**Fig. 6:**
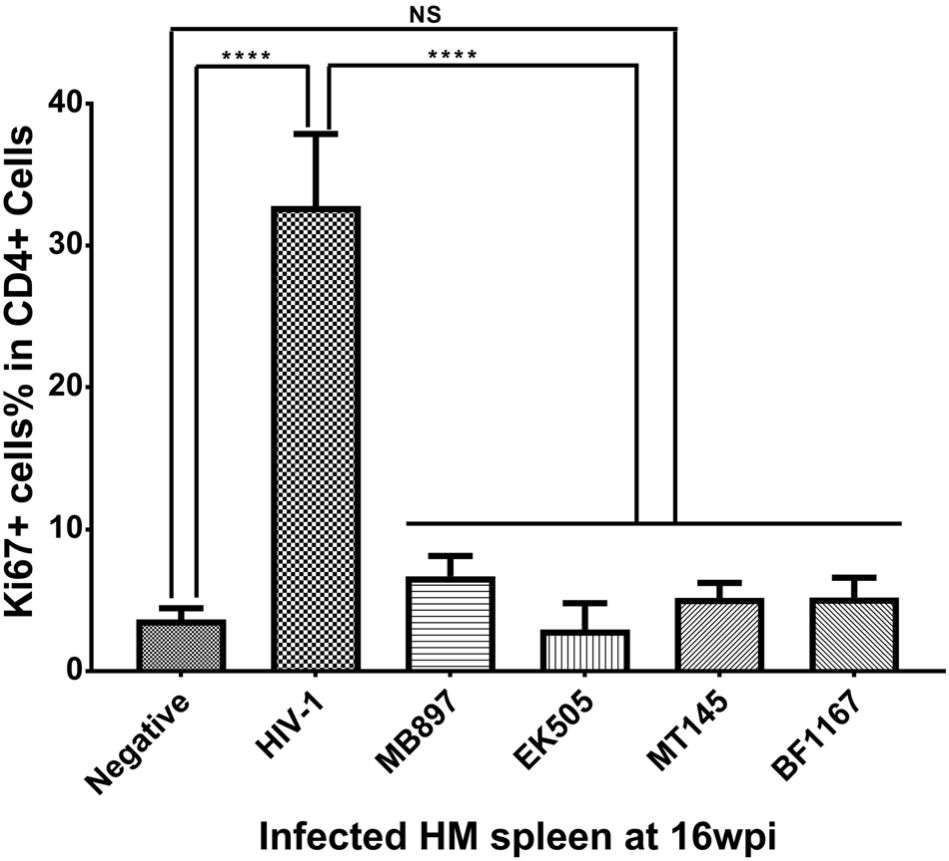
Quantification of the percentage of Ki67+ cells within CD4+ T cells in splenic tissues of SIVcpz- and HIV-1-infected hu-BLT mice after immunofluorescent co-staining of human CD4 and Ki67. Statistical significance is indicated with stars. NS non-significance, ***<0.001

Immune activation is another hallmark of HIV-1 disease progression; therefore, we compared CD4+ T-cell immune activation as a parameter of pathogenicity between SIVcpz- and HIV-1-infected hu-BLT mice. We quantified immune activation of peripheral blood CD4+ T cells using flow cytometry. Figure 4 shows the CD4+ T-cell activation kinetics based on the percentage of HLA-DR+ CD38+ cells in CD4+ T cells throughout the 16 weeks of infection. Compared with the HIV-1-infected group, CD4+ T-cell activation in the SIVcpz-infected group was significantly lower (*p* < 0.001) at all time points post-infection. When compared to the uninfected control group, there was no significant difference in CD4+ T-cell activation in the SIVcpz-infected group at all time points. We also quantified immune activation in the secondary lymphatic tissues of SIVcpz- and HIV-1-infected hu-BLT mice using immunofluorescent staining of human ki67 and human CD4. As shown in Fig. 5, there were many more CD4+ T cells and fewer Ki67+ cells in the SIVcpz-infected groups and uninfected control group compared with the HIV-1-infected group. The percentage of Ki67+ cells in CD4+ T cells was significantly higher in HIV-1-infected splenic tissues than in uninfected (*p* < 0.001) and all SIVcpz-infected tissues (*p* < 0.001) (Fig. 6). There were no significant differences between different SIVcpz strains and SIVcpz-infected versus uninfected splenic tissues (*p* > 0.05) (Fig. 6).

We therefore concluded that infection with SIVcpz strains that are closely related to ancestral HIV-1 results in significantly less or no immune activation and CD4+ T-cell deletion compared with HIV-1 infection, despite having similar levels of plasma viral load.

### Comparative transcriptome analysis of CD4+ T cells during SIVcpz and HIV-1 infection

**Fig. 7:**
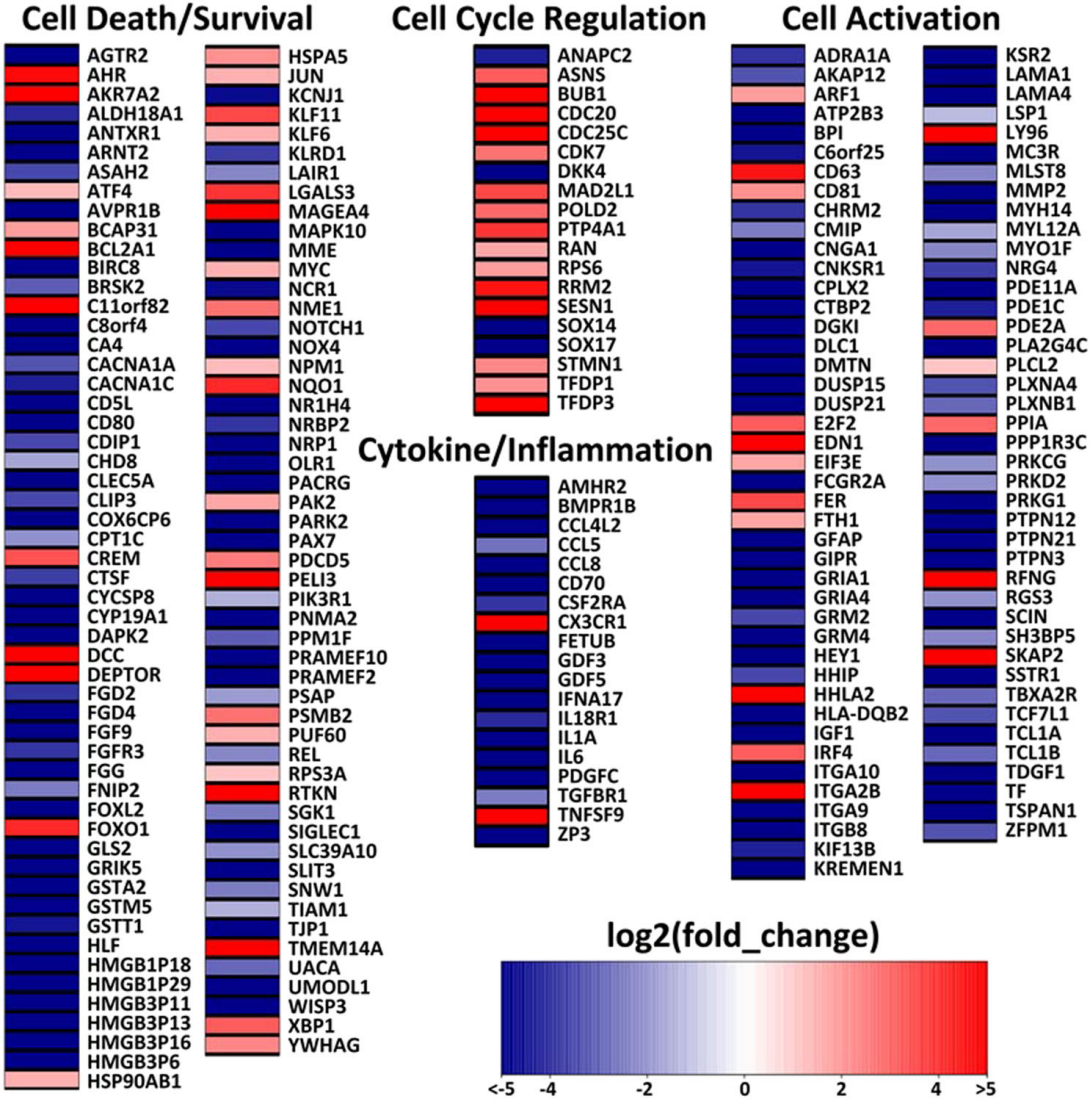
Functional transcriptome comparison of human CD4+ T cells from spleen tissues of SIVcpz- and HIV-1-infected hu-BLT mice. Heat map of differentially expressed genes (DEGs) in SIVcpz-infected animals compared with HIV-1-infected animals. Log2 fold change is color coded with a range from less than −5 to greater than 5

To further understand the underlying mechanism of the contrasting pathogenicity of SIVcpz- versus HIV-1-infected hu-BLT mice, CD4+ T cells were isolated from splenic tissues of SIVcpz-infected (*n* = 3 for SIVcpzMB897 infection, *n* = 3 for BF1167 infection) and HIV-1-infected (*n* = 3) animals at 16 wpi using negative magnetic cell sorting. Both the CD4+ T-cell number and purity isolated from splenic tissues were normalized between the SIVcpz and HIV-1 infection groups. RNA was extracted from the isolated cells for mRNA-Seq. Both SIVcpzMB897 and SIVcpzBF1167 had similar pathogenicity profiles in terms of CD4+ T-cell counts and immune activation, so we compared both SIVcpz-infected animals with HIV-1-infected animals. As a result, 1448 differentially expressed genes (DEGs) were identified from a total of 16,210 genes with measured expression using a threshold of 0.0678 (adjusted *Q*-value) for statistical significance and an absolute value of log2 fold change greater than 2. We focused on analyzing DEGs in the categories of cell death and survival, cell activation, cytokine/inflammation, and cell cycle regulation, corresponding to the observed differences in CD4+ T-cell death/survival and immune activation between SIVcpz and HIV-1 infections. Figure 7 shows a heat map of DEGs in SIVcpz-infected animals compared to HIV-1-infected animals. In the cell death or survival category (106 DEGs), more than 90% of DEGs follow the pattern of decreasing expression level of genes related to cell death and increasing expression level of genes related to cell survival in SIVcpz-infected animals compared to HIV-1-infected animals. Nineteen DEGs that code for protective proteins that promote cell survival or protect against cell death were upregulated, including AHR^25,26^, AKR7A2^27^, ATF4^28,29^, BCAP31^30–32^, BCL2A1^33^, C11orf82^34^, DEPTOR^35^, HSP90AB1^36^, HSPA5^36^, JUN^37^, KLF6^38^, MAGEA4^39^, NQO1^40^, PAK2^41^, RPS3A^42,43^, RTKN^44^, TMEM14A^45^, XBP1^46^, and YWHAG^47^. In the cell cycle regulation category (19 DEGs), only four DEGs, ANAPC2, DKK4, SOX14, and SOX17, had decreased expression levels in SIVcpz-infected animals compared to HIV-1-infected animals. ANAPC2 prevents the pre-mature entry of S phase and triggers mitotic exit^48^. DKK4 and SOX17 are Wnt inhibitors that prevent cell cycle progression or proliferation^49,50^. Sox14 overexpression was reported to induce apoptosis^51^. Reports have previously shown that apoptosis goes hand in hand with cell cycle arrest at G2/M in HIV-infected cells^52–54^. Almost all the DEGs in the cell cycle regulation category followed a pattern of prompting cell cycle progression and halting cell cycle arrest in SIVcpz-infected animals. For the cytokine and inflammatory genes category (19 DEGs), all the DEGs except for two displayed a pattern of decreasing cytokine/cytokine receptor interaction and decreasing proinflammatory responses in SIVcpz-infected animals compared with HIV-1-infected animals. Furthermore, the cytokine/cytokine receptor pathway (*p* = 0.004) was significantly downregulated in SIVcpz-infected animals compared with HIV-1-infected animals. For the cell activation category (84 DEGs), 80% of DEGs followed a pattern of decreasing cell activation in CD4+ T cells in SIVcpz-infected animals compared with HIV-1-infected animals. We also performed transcriptome analysis by comparing SIVcpzMB897 vs. HIV-1 and SIVcpzBF1167 vs. HIV-1. Both SIVcpz viruses had similar transcriptome profiles, and individual comparisons yielded comparable results to the combined transcriptome analysis of SIVcpz vs. HIV-1. Thus, the transcriptome analysis results were consistent with the minimal CD4+ T-cell depletion and immune activation in SIVcpz-infected animals observed using flow cytometry and IHCS/QIA. Transcriptome analysis revealed that SIVcpz-infected animals had lower expression levels of genes related to cell death, cell cycle arrest, cytokine/cytokine receptor interactions, inflammatory responses, and cell activation, as well as higher expression levels of genes related to cell survival and promotion of the cell cycle, thus providing critical mechanistic clues for the differences in pathogenicity observed between SIVcpz- and HIV-1-infected animals.

## Discussion

The HIV-1 pandemic began in the early 1980s as the consequence of the cross-species transmission of SIVcpz to humans^1–3^, resulting in one of the most devastating infectious diseases in recorded human history. Although many hypotheses have been proposed to explain the evolutionary origins of HIV-1, no experimental evidence exists for evaluating or recapitulating the initial pathogenicity of SIVcpz to humans after crossing the species barrier. The long period of clinical “silence” between the estimated time of SIVcpz cross-species transmission and the first documented human HIV-1 group M infection in the Congo^9^ calls for further study. The date of the MRCA of the shared HIV-1 group M and SIVcpz was estimated to be around 1853^6^ or 1876^7^. Thus, the time of cross-species transmissions of the ancestral group M virus-SIVcpz to humans is very distant. Although isolated and self-limited SIVcpz pathogenic infections of humans may explain the delayed pandemic infection, a plausible hypothesis can be that SIVcpz initial infections of humans were less pathogenic or non-pathogenic, with the virus gaining pathogenicity over time in the human population. Furthermore, there is evidence showing continuing cross-species transmission of SIV from monkeys^10,11^ and great apes^4,12^ to humans. Thus, further evaluation of the pathogenicity of SIVcpz may be a worthwhile endeavor to enhance our ability to predict future spill-over events in addition to better understand the evolutionary origins of the HIV-1 pandemic.

To better understand the evolutionary history of HIV-1 and to predict the risk of emergence of another HIV-1-like infectious disease in humans in the future, it is fundamental to assess the in vivo pathogenesis of SIVcpz to humans. However, for clear ethical reasons, a study cannot be directly performed in humans. Hu-BLT mice are the best available in vivo model of the human immune system, which has been successfully applied in recapitulating the major events of HIV-1 pathogenesis in humans, such as CD4+ T-cell depletion and generalized immune activation^14,15,18,55^. We previously studied the infectivity and transmissibility of multiple SIVcpz strains to humans using hu-BLT mice^18^. Herein, we further investigated the pathogenicity of several SIVcpz strains in humans using this model. We found that the strains of SIVcpz, including the SIVcpz strains that are closely related to the ancestral viruses of the HIV-1 groups M (SIVcpzMB897) and N (SIVcpzEk505) and two lineages of SIVcpz that are not associated with any known HIV-1 infections in humans (SIVcpzMT145 and SIVcpzBF1167), are less pathogenic or non-pathogenic to humans compared with HIV-1 infection. There is significantly less or no CD4+ T-cell depletion and immune activation in SIVcpz-infected animals compared with HIV-1-infected animals despite similar plasma viral loads. CD4+ T-cell decline in the peripheral blood of hu-BLT mice infected with HIV-1 was observed a few weeks post-HIV-1 infection, which is consistent with previous reports. The rapid kinetics of CD4+ T-cell depletion observed in hu-BLT mice after HIV-1 infection are different from those after human HIV-1 infection, and it was estimated that 2.6 days in adult mice is equivalent to 1 human year^56^. In concordance, transcriptome analysis revealed that SIVcpz-infected animals had lower expression levels of genes related to cell death, cell cycle arrest, cytokine/cytokine receptor interactions, inflammatory responses, and cell activation as well as higher expression levels of genes related to cell survival and promotion of the cell cycle. CD4+ T-cell depletion and immune activation are the hallmarks of human HIV-1 infection and disease progression^57,58^. Therefore, our findings show that there is pathogenic discrepancy between SIVcpz strains that are closely related to ancestral HIV-1 and HIV-1.

We would like to note that the SIVcpz strains used in this study are proximate and may not truly represent the SIVcpz that initiated that pandemic HIV-1 infection, as it is impossible to ascertain which SIVcpz strains are the true ancestral HIV-1. However, our studied SIVcpz strains are closely related to the ancestral strains of HIV-1 groups M and N.

This study is the first to clearly show that SIVcpz strains that are closely related to the ancestral viruses of HIV-1 groups M and N, as well as the lineages of SIVcpz that are not associated with any known HIV-1 infections in humans, are less pathogenic or non-pathogenic compared with HIV-1 in a hu-BLT mouse model. This could be analogous to reported cases of HIV-infected non-progressors who had high viremia but limited CD4 T-cell depletion^59,60^. Our findings suggest that SIVcpz or ancestral HIV-1 virus may be less pathogenic than HIV-1 in humans and support a new model for the evolutionary origins of HIV-1, where the initial SIVcpz cross-species transmission virus may initially be less pathogenic or non-pathogenic and gains virulence over time in the human population. Future studies are needed to elucidate the molecular foundation for the differences in pathogenicity observed in this study between HIV-1 and SIVcpz.
